# Non-operative management of blunt abdominal trauma. Is it safe and feasible in a district general hospital?

**DOI:** 10.1186/1757-7241-17-22

**Published:** 2009-05-13

**Authors:** George A Giannopoulos, Iraklis E Katsoulis, Nikolaos E Tzanakis, Panayotis A Patsaouras, Michalis K Digalakis

**Affiliations:** 1 1st Surgical Department, "Asklepieion Voulas" General Hospital, 1 Vasileos Pavlou str, 166 73, Athens, Greece

## Abstract

**Background:**

To evaluate the feasibility and safety of non-operative management (NOM) of blunt abdominal trauma in a district general hospital with middle volume trauma case load.

**Methods:**

Prospective protocol-driven study including 30 consecutive patients who have been treated in our Department during a 30-month-period. Demographic, medical and trauma characteristics, type of treatment and outcome were examined. Patients were divided in 3 groups: those who underwent immediate laparotomy (OP group), those who had a successful NOM (NOM-S group) and those with a NOM failure (NOM-F group).

**Results:**

NOM was applied in 73.3% (22 patients) of all blunt abdominal injuries with a failure rate of 13.6% (3 patients). Injury severity score (ISS), admission hematocrit, hemodynamic status and need for transfusion were significantly different between NOM and OP group. NOM failure occurred mainly in patients with splenic trauma.

**Conclusion:**

According to our experience, the hemodynamically stable or easily stabilized trauma patient can be admitted in a non-ICU ward with the provision of close monitoring. Splenic injury, especially with multiple-site free intra-abdominal fluid in abdominal computed tomography, carries a high risk for NOM failure. In this series, the main criterion for a laparotomy in a NOM patient was hemodynamic deterioration after a second rapid fluid load.

## Background

In the early 70s, Singer *et al. *[1] described the incidence and mortality of overwhelming post-splenectomy infection (OPSI) in 2795 asplenic patients. The preservation of the spleen was initially applied in pediatric trauma and later in adults with excellent results. Advances in medical imaging and minimally invasive techniques have highly contributed to the extension of non-operating management (NOM) in more severe, complex, even penetrating injuries. Currently, NOM is considered as standard of care in all hemodynamically stable injured adults without peritoneal signs and numerous recent studies demonstrate success rates exceeding 80% [2-6].

NOM of liver injuries has an even higher success rate, exceeding 90% [3]. Velmahos *et al.*[7] support that the liver is a sturdy organ and conclude that in the absence of peritoneal signs and irreversible instability, all liver injuries can be treated conservatively regardless the magnitude of injury. NOM is also very successful in renal injuries with success rates over 90% [4]. On the contrary, NOM in pancreas trauma is still limited and problematic [3].

Most studies concerning NOM were designed and carried out in specialized hospitals (level I trauma centers) with dedicated human resources, surgical/trauma ICU and extensive minimally invasive or endoscopic facilities. In the present study, NOM was attempted in a district general hospital with shortage of ICU beds, surgical staff and fellows not exclusively working on trauma and limited access to percutaneous or endoscopic techniques.

## Methods

This is a prospective study including 30 patients with blunt abdominal trauma that have been treated in the 1^st ^Surgical Department of "Asklepieion Voulas" Hospital between July 2006 and December 2008. All stable or responding unstable patients without peritoneal signs were treated non-operatively, regardless the organ or the grade of injury. Focused abdominal sonography for trauma (FAST) and abdominal computed tomography (CT) with iv contrast was performed in all stable patients, whilst hypotensive ones were examined only with FAST. Categorization of patients as "stable" or "responders", as well as resuscitation in emergency department (ED) was according to ATLS^® ^[8] guidelines. In every patient with hypotension (<90 mmHg) and tachycardia (>100 p/min), 2.000 ml of intravenous fluids were rapidly administered. The patients that showed hemodynamic improvement in ED, even when a mild tachycardia (<110 p/min) persisted, were considered as "responders". Patients who were admitted with hypotension and tachycardia, deteriorated despite resuscitation, had a positive FAST and no other obvious site of bleeding, underwent emergency laparotomy. This was also the case in those who responded transiently and relapsed in ED. Patients who died in ED were excluded. The survivors were divided in 3 groups: those who were operated immediately (OP), those who had a successful NOM (NOM-S) and those in whom NOM failed (NOM-F). Laparotomy to a patient who left ED with a decision for NOM was considered as failure regardless the time interval. The non-ICU patients were hospitalized on the surgical ward connected to monitor device and palmic oxymeter. The decision to operate a NOM patient was mainly based on deterioration of the hemodynamic status, after another fluid load (2000 ml). The rationale was that transient tachycardia or hypotension could occur from a non-abdominal origin (e.g. extra-abdominal trauma, medication, inadequate volume replacement) or from an ongoing, but modest intra-abdominal bleeding.

In our study, there was not a cut-off hematocrit value and therefore transfusion was rather empirical. However, older patients (>70 years old) and patients with coronary disease were transfused when hematocrit was lower than 30% (or hemoglobin <10 dl/ml), even if they were stable. The attending surgeon was in-charge concerning patient's management. No patient from this series was transferred to a higher-level trauma center in an acute setting.

**Figure 1:**
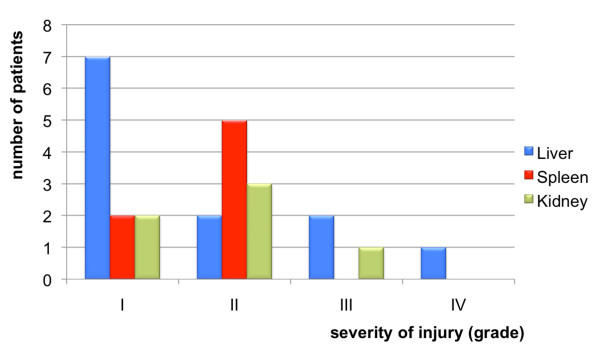
**Organ-specific severity of injury in NOM group**. Note that a patient may have injury in more than one organ.

The recorded patients' data were age, sex, medical history (comorbidities) and mechanism of injury (Table 1). Relevant comorbidities were hypertension, coronary disease, heart failure, chronic obstructive pulmonary disease and diabetes mellitus. Mechanism of injury was defined either as road traffic accident (RTA) or non-RTA, which included all the remaining mechanisms. Trauma severity was evaluated according to *Injury Severity Score *(ISS) and organ injury according to *Injury Scaling and Scoring System *[9]. Patients' status in admission was evaluated by ISS, admission hematocrit, hemodynamic stability (SBP >90 mmHg, PR <100), intubation in ED and FAST findings (Table 1). Since those who required transfusion in the first hour were operated, the need for transfusion was evaluated in the first 24 hours. Isolated (liver, spleen, kidney), multiple solid organ abdominal ones and severe extra-abdominal injuries were recorded (Table 2). Length of stay, morbidity and mortality were examined (Table 3). Organ-specific severity of injury in NOM group was also evaluated (Figure 1)

**Table 1:** Demographic and admission characteristics

	***NOM-S group*** (n = 19)	***OP group*** (n = 8)	***NOM-F group*** (n = 3)	***P***^††^
Age^†^	31 (15 – 80)	28 (21–71)	63 (37–69)	*0.815
Male sex	11 (58%)	7 (88%)	0	0.124
Comorbidities	5 (26%)	2 (25%)	2 (66.7%)	0.891
RTA	17 (90%)	6 (75%)	3 (100%)	0.332
ISS^†^	16 (6 – 41)	43 (21 – 75)	29 (14–29)	* <0.001
Admission Hct (ED)^†^	41 (32 – 46)	27 (24 – 49)	40 (39–43)	*0.008
SBP >90 mmHg (ED)	18 (95%)	1 (13%)	2 (66.7%)	**<0.001**
PR < 100 (ED)	15 (79%)	0 (0%)	2 (66.7%)	**<0.001**
RBC (IU) (first 24 h)^†^	0 (0 – 3)	6 (0 – 11)	0 (0–2)	* <0.001
Intubation (ED)	1 (5%)	4 (50%)	0	**0.006**
Positive FAST	13 (68%)	8 (100%)	3 (100%)	0.072

RTA, Road Traffic Accident; ISS, Injury severity score; ED, emergency department; SBP, systolic blood pressure; PR, pulse rate

*Mann Whitney *U *test and *χ*^2^, ^†^Median and range, ^††^Comparison between NOM-S and OP group

**Table 2:** Injury characteristics

	***NOM-S group*** (n = 19)	***OP group*** (n = 8)	***NOM-F group*** (n = 3)	***P***^†^
Liver injury	12 (63%)	6 (75%)	0	0.482
Splenic injury	7 (37%)	6 (75%)	3 (100%)	0.068
Kidney injury	3 (16%)	3 (38%)	0	0.227
Multiple solid abdominal organ injury	3 (16%)	5 (63%)	0	**0.012**
Extra-abdominal injury	16 (84%)	8 (100%)	3 (100%)	0.215

^†^Comparison between NOM-S and OP group

**Table 3:** Type, length of hospitalization and outcome

	***NOM-S group*** (n = 19)	***OP group*** (n = 8)	***NOM-F group*** (n = 3)	***P***^††^
ICU admission	1 (5%)	4 (50%)	0	**0.006**
LOS^†^	6 (2 – 12)	17 (1–187)	9 (8–10)	*0.018
Morbidity	0	3 (37.5%)	0	**<0.001**
Mortality	0	3 (37.5%)^§^	0	**<0.001**

LOS, length of stay (days)

^†^Median and range, ^††^Comparison between NOM-S and OP group, *Mann Whitney *U *test and *χ*^2^, ^§^Two of these 3 patients died on the operating table

### Statistical analysis

OP and NOM-S groups were compared using *χ*^2 ^test for categorical variables and Mann Whitney *U *test for continuous ones. The sample size (n = 3) of NOM-F group was insufficient in order to perform statistical comparison with NOM-S group. Analysis was performed using SPSS 12.0.1 for Windows (SPSS Inc. Chicago, IL, USA). All statistical tests were performed at a = 0.05 significance level.

## Results

Non – operative management was initially applied in 73.3% (22 patients) of all blunt abdominal injuries with a failure rate of 13.6% (3 patients). No significant differences were observed between OP and NOM-S group in relation with age, sex, comorbidities, extra-abdominal trauma and mechanism of injury (Table 1). On the contrary, in NOM-S group significantly fewer patients were intubated in ED and presented with hypotension and tachycardia. They also had a significantly lower ISS, higher admission hematocrit and lower need for transfusion. One patient from NOM-S group was intubated upon arrival to the ED and subsequently transferred to the ICU for ventilation support due to multiple rib fractures. The concomitant grade II splenic injury was treated conservatively.

In the OP group, significantly more patients had injury in multiple solid abdominal organs and the most commonly associated organ was the spleen, albeit with marginal significance (*P *= 0.068). Two patients (with ISS: 75) died on the operating table due to non-reversible hemorrhagic shock. Another patient who was transferred to the ICU postoperatively had a complicated course, underwent 3 reoperations for abdominal collections and enterocutaneous fistulae; developed abdominal compartment syndrome and finally died 6 months later. Three other patients were transferred postoperatively to the ICU, one of whom developed Adult Respiratory Distress Syndrome (ARDS) and two were successfully re-operated in order to drain abdominal collections.

NOM failed in three cases, all female. Two of them had splenic injury (grade II and III, respectively) and finally underwent laparotomy (on the 1^st ^and 4^th ^post-admission day, respectively) due to hemodynamic instability. The third patient sustained a minor (grade I) splenic trauma but CT revealed free intra-abdominal fluid in multiple sites. Six hours later she was operated and a mesenteric laceration was found. NOM-F group was not considered adequate for statistical evaluation. However, these patients in comparison with NOM-S were older, with more comorbidities and suffered mainly from splenic injury. Two of 3 (66.7%) were stable in ED and all of them had a normal initial hematocrit. Even so, FAST was positive in all cases.

## Discussion

In the present study, hemodynamic status, admission hematocrit, need for transfusion and ISS were significantly different between OP and NOM-S group. Most reports conclude that the first three characteristics are significant predictors of NOM success. Nevertheless, all the NOM-F patients had a normal initial hematocrit. Moreover, it seems that there is not a definite and clear limit for transfusion. Some authors report that in cases with splenic trauma requiring more than 1 UI RBC, NOM is likely to fail. Others, especially for non-splenic trauma, suggest a 4 IU limit [6]. In our series, there was not a specific protocol concerning transfusion and blood was given empirically guided by the hemodynamic status. Controversy exists in the prognostic value of ISS and Glasgow Coma Scale (GCS). Furthermore, the term *hemodynamic instability *and especially the state of *responding instability *are still ambiguous [4,7,10]. This arbitrary cut-off point seems to be critical in decision of laparotomy and Harbrecht *et al. *[11] support that this is a major factor not only for NOM failure but also for preventable deaths. Our basic criterion in operating a NOM patient was deterioration of hemodynamic status, despite a second attempt for resuscitation.

The majority of authors concur that the associated organ is important, even decisive in NOM success. The non-splenic blunt injury has been identified as independent prognostic factor. Moreover, splenic trauma is reported to have the highest failure rates, reaching 30% [6,12]. Yanar *et al. *[6] estimate that 50% of failure cases were due to the spleen. In our study, the associated organs in NOM-S group were the liver (63%), the spleen (37%) and the kidney (16%). Conversely, splenic trauma was present in 75% of cases in the OP group. In addition, 2 of 3 (66.6%) NOM-F cases were splenic injuries.

An important issue concerning spleen preservation is prevention of OPSI. Nevertheless, the lifetime risk for death from OPSI following traumatic splenectomy in adults does not exceed 0.02% [13]. Therefore, it seems that the risk for death from striving to preserve the spleen in unstable patients is inordinately higher than death risk from OPSI [13].

Liver has proven to be a sturdy and durable organ as the vast majority of the cases are being treated conservatively. In the present study, none of the NOM-F cases was due to hepatic hemorrhage. Although bleeding-associated mortality does not seem to be the main concern, some authors stress that grade IV and V liver injuries are often associated with high morbidity (21% and 63%, respectively). The majority of such complications as ongoing bleeding, biloma, bile peritonitis, abscess or fistulae can be successfully treated with selective angioembolism, percutaneous drainage, ERCP and other minimally invasive procedures [7,14].

In the present study, multiple solid abdominal organ injury demonstrated a significant difference between OP and NOM-S group, but was not present in any NOM-F patient. Although, multiplicity of injury was traditionally associated with higher failure rates, recent studies show opposite results [6]. Shortage of certain supportive means, such as ICU beds, possibly facilitates a "preventive" operation. Nevertheless, in the present series the majority of patients of the NOM-S group remained on the ward under close monitoring and only one patient who was intubated upon arrival to the ED due to multiple rib fractures and remained in the ICU where his grade II splenic injury was treated conservatively. Similar observations were reported by a study from Israel, a country with population comparable to ours [10].

FAST is currently the mainstay in initial assessment of trauma, but abdominal CT with iv contrast is imperative in order to proceed in NOM. Furthermore, Salim *et al. *[15] examined the value of whole body imaging (pan scan) in blunt trauma without obvious signs of injury and concluded that this approach changed planned treatment in 19% of cases. Although findings were not always concerning life-threatening injuries, they allowed earlier discharge. Delayed-phase CT findings and the amount of free intra-abdominal fluid (more than 300 ml) have been described as independent prognostic factors for NOM success [4,16,17]. Volumetric assessment is not always feasible but free fluid detected in more than two sites is highly predictive of failure. This was the case in a NOM-F patient with grade I splenic injury but multiple site free fluid due to laceration of mesentery.

NOM was initially applied in 73.3% of blunt abdominal injury and the overall success rate, regardless the organ involved, was 13.6%. Without doubt, these results are not directly comparable to other studies as the injury grade distribution varies among studies. Besides, decision to operate does not only depend on the clinical status of the patient, for which no clear guidelines have been described, especially in the "gray zone". Personal judgment and experience, hospital's infrastructure and homogeneity of the team are important, often decisive factors. According to our findings, we consider that NOM is feasible in a middle volume general hospital but constant awareness and early identification of "gray zone" patients is critical in order to reduce morbidity and preventable deaths.

## Conclusion

NOM of blunt abdominal trauma is not a novelty, but in a district hospital's environment is often a challenge. Our limited experience showed that laparotomy is probably the most reasonable choice in persistent or borderline hemodynamic instability due to splenic trauma, especially in shortage of supportive means. Moreover, free abdominal fluid in multiple sites is a sign of a possible NOM failure, even when abdominal CT reveals minor solid organ injury. The hemodynamically stable or easily stabilized trauma patient can be admitted in a non-ICU ward, with the provision of close monitoring.

## Competing interests

The authors declare that they have no competing interests.

## Authors' contributions

GAG was involved in conception, design, analysis and interpretation of data; drafting the manuscript. IEK was involved conception and design, acquisition, analysis and interpretation of data; performed statistical analysis; revision of the manuscript. NET was involved in acquisition of data and drafting the manuscript. PAP was involved in acquisition of data and drafting the manuscript. MKD was involved in coordination of the study and revision of the manuscript. All authors read and approved the final manuscript
